# ­­­Successful Application of Whole Cell Panning for Isolation of Phage Antibody Fragments Specific to Differentiated Gastric Cancer Cells

**DOI:** 10.15171/apb.2019.072

**Published:** 2019-10-24

**Authors:** Sepideh Nikfarjam, Mohammad Reza Tohidkia, Tayebeh Mehdipour, Ramin Soleimani, Ali Akbar Rahim Rahimi, Mohammad Nouri

**Affiliations:** ^1^Research Center for Pharmaceutical Nanotechnology, Tabriz University of Medical Sciences, Tabriz, Iran.; ^2^Department of Medical Biotechnology, Faculty of Advanced Medical Sciences, Tabriz University of Medical Sciences, Tabriz, Iran.; ^3^Department of Molecular Biology, Research and Diagnostic Laboratory of Dook, Sari, Iran.; ^4^Department of Microbiology, Faculty of Medicine, Tabriz University of Medical Sciences, Tabriz, Iran.; ^5^Stem Cell and Regenerative Medicine Institute, Tabriz University of Medical Sciences, Tabriz, Iran.; ^6^Department of Biochemistry and Clinical Laboratories, Faculty of Medicine, Tabriz University of Medical Sciences, Tabriz, Iran.

**Keywords:** Gastric cancer, Phage display, Proteomics, scFv, Tumor targeting, Whole cell panning

## Abstract

***Purpose:*** Generation of antibodies which potentially discriminate between malignant and healthy cells is an important prerequisite for early diagnosis and treatment of gastric cancer (GC). Comparative analysis of cell surface protein landscape will provide an experimental basis for biomarker discovery, which is essential for targeted molecular therapies. This study aimed to isolate phage-displayed antibody fragments recognizing cell surface proteins, which were differently expressed between two closely related GC cell lines, namely AGS and MKN-45.

***Methods:*** We selected and screened a semisynthetic phage-scFv library on AGS, MKN-45, and NIH-3T3 cell lines by utilizing a tailored selection scheme that was designed to isolate phagescFvs that not only recognize the differentiated AGS cells but also distinguish them from NIH3T3 fibroblasts and the poorly differentiated MKN-45 cells.

***Results:*** After four rounds of subtractive whole cell panning, 14 unique clones were identified by ELISA screening and nucleotide sequencing. For further characterization, we focused on four phage-scFvs with strong signals in screening, and their specificity was confirmed by cell-based ELISA. Furthermore, the selected phage-scFvs were able to specifically stain AGS cells with 38.74% (H1), 11.04% (D11), 76.93% (G11), and 69.03% (D1) in flow cytometry analysis which supported the ability of these phage scFvs in distinguishing AGS from MKN-45 and NIH-3T3 cells.

***Conclusion:*** Combined with other proteomic techniques, these phage-scFvs can be applied to membrane proteome analysis and, subsequently, identification of novel tumor-related antigens mediating proliferation and differentiation of cells. Furthermore, such antibody fragments can be exploited for diagnostic purposes as well as targeted drug delivery of GC.

## Introduction

Gastric cancer (GC) was the fourth most prevalent tumor and the third leading cause of cancer-associated mortality worldwide in 2017.^1^ Poor clinical outcomes of GC is attributed to the scarcity of suitable antigen(s) which is required for early diagnosis and therapy. Comprehensive knowledge about the molecular context of cancerous cells and identification of novel targets and therapeutics can open new avenues for treatment of GC.^2,3^ Proteomics offers high potential for identification of disease-related biomarkers by comparing the proteome of a diseased cell/tissue with the proteome of the respective non-diseased cells/tissues.^4^ In this respect, high-throughput screening of combinatorial antibody libraries can be employed for isolation of membrane-specific antibodies which enable us to detect cell surface proteins differently expressed between tumor and non-tumor cells.^5,6^

Phage display technology (PDT) provides a powerful screening tool to isolate functional antibody fragments with binding affinity for every possible target protein. Antibody fragments represent one of the most important classes of specificity molecules with small antigen binding sites that recognize antigens with the same affinities and specificities as conventional antibodies. Two typical forms of these proteins are scFv (single chain fragment variable) and Fab (fragment antigen binding). In comparison with hybridoma-derived antibodies, antibody fragments are considered more appropriate probes in antibody arrays for analyzing thousands of proteins in parallel due to their rapid production and manipulation.^7^ The integration of PDT with proteomics can be used for simultaneous identification of disease-related biomarkers and the discovery of therapeutic antibodies.^8^ In phage antibody libraries, antibody variable regions are ligated in-frame to a filamentous phage coat protein gene in a phagemid vector. After transformation into a proper *E. coli* host, the antibody fragment-displaying phage particles are produced through infection with a helper phage and prepared for the selection process known as ‘panning’. Panning is the successive rounds of selection which specifically enriches candidate binders with desired properties via incubation of library with the target antigen, washing out the non-specific binders, elution to retrieve the specific binders, and finally, amplification in bacteria to prepare for the next round of selection. Isolation of specific antibody clones will provide access to the antibody-encoding genes. Based on the intended application, various types of panning methods have been so far employed such as solid-phase selection on an immobilized purified antigen, solution-phase selection with a biotinylated antigen, and whole cell panning (WCP) using prokaryotic or mammalian cells. Mammalian WCP utilizes intact cells in monolayers or suspension for selection of antibodies against the native three-dimensional structure of membrane antigens in the presence of a lipid bilayer.^9^ Therefore, WCP will result in biologically relevant binders that can identify naturally exposed epitopes.^10-14^ In contrast, the expression of membrane proteins in aqueous media, in both solid and solution phase selections may cause conformational alterations and/or aggregation, and consequently, the binders may recognize the epitopes that are naturally masked in the native form. However, WCP is practically problematic and often associated with enrichment of binders to unwanted common cell surface epitopes, due to complex antigenic context of cellular membrane, and low abundance and limited exposure of membrane proteins.^15^ To overcome this drawback, tailored subtraction methods were extensively exploited and the libraries were selected on the intended cancer cells preceded with a depletion on equivalent healthy cells.^2,4,16-19^

In the current study, we utilized a subtractive WCP scheme to isolate phage-scFvs capable of specifically recognizing the differentiated gastric adenocarcinoma cell line. For this purpose, we panned a human single-fold library against live AGS cells in suspension with a prior depletion on NIH-3T3 and MKN-45 cells, respectively representative of healthy and poorly differentiated cell lines, to remove the binders to common surface proteins.

## Materials and Methods

### Cell culture

AGS and MKN-45 (human gastric adenocarcinoma cell lines) and NIH-3T3 (murine fibroblasts) cell lines were acquired from Iranian Biological Resource Center (IBRC, Tehran, Iran). All cell lines were authenticated by STR (Short Tandem Repeat) profiling at the Human and Animal Cell Bank of IBRC and regularly tested for mycoplasma contamination.^3^ Gastric cell lines were cultivated in RPMI-1640 (Sigma-Aldrich, St. Louis, MO, USA) supplemented with 10% or 20% fetal bovine serum (FBS) (Gibco, Carlsbad, CA, USA) for AGS and MKN-45 cells, respectively. NIH-3T3 cells were cultured in DMEM (Gibco) containing 10% FBS. All cells were maintained at 37°C under a humidified atmosphere of 5% CO_2_ air and regular subculture was done every 3-5 days with 0.25% trypsin-EDTA (Gibco).^20^

### Phage library and bacterial strains

Human single-fold semisynthetic phage-scFv library (Tomlinson I + J), *E. coli* strains: TG1 (T-phage resistant) and HB2151, and KM13 helper phage were obtained from Source BioScience (Nottingham Business Park, Nottingham, UK).^21,22^ The library was constructed by the insertion of scFv-encoding genes approximate to gIII in a phagemid vector containing ampicillin resistance marker (pIT2) and transformed into TG1 strain. The amber stop codon located between scFv and gIII sequences allows for either the display of scFv-pIII fusion proteins in the suppressor strain TG1 or production of free soluble scFvs in the non-suppressor strain HB2151.^23,24^ KM13 helper phage containing the kanamycin resistance gene was used for the library rescue.

### Selection on live cells

Each round of library selection consisted of two depletion steps with NIH-3T3 and MKN-45 as negative target cells and a positive selection on AGS cells. The recombinant scFv-displaying phages were rescued with 2x10^11^ PFU (plaque forming unit) of KM13 helper phage as described by the library manual instructions.^25^ All incubation steps were carried out at 4°C for 1 h with gentle shaking in a total volume of 5 ml. The cells were grown to 70% confluency, washed, and harvested by cell dissociation buffer (Gibco). Single cell suspensions were normalized to a concentration of 2×10^6^ cells per ml and blocked in freshly prepared PBS-3% BSA (w/v; phosphate buffered saline containing 3% bovine serum albumin) (Sigma-Aldrich).^24^ Concurrently, an aliquot of freshly rescued phage-scFv library, 10^12^ CFU (colony forming unit), was blocked on an overhead rotator. The blocked phage-scFvs were depleted using the negative target cells. Following centrifugation at 500 g for 10 min, the supernatant was put through the positive selection. Afterward, the cell pellets were washed 5-10 times with PBS and AGS-binding phage-scFvs were eluted by resuspension in trypsin solution (0.1 mg/mL). The eluate was used to infect exponentially growing *E. coli* TG1 at OD_600_ (optical density) of 0.4 to propagate phages for the next round of selection. Subsequent titration of the eluted phage particles, phage rescue and production were carried out as described elsewhere.^25-27^

### Polyclonal phage enzyme-linked immunosorbent assay (ELISA)

The enrichment of AGS-specific binders was verified by polyclonal phage ELISA.^27^ The amplified phages after each selection round were tested for binding to AGS and MKN-45 cells. The cells were detached as previously described and blocked in 2% (w/v) skimmed milk (Merck, Kenilworth, NJ, USA) in PBS and distributed approximately 5x10^5^ cells/well in a 96-well flat-bottomed tissue culture plate (SPL Life Sciences, Gyeonggi-do, South Korea).^28^ The blocked phages were allowed to bind to the cells for 90 min at 4°C. Followed by several washing steps, the cell surface-binding phage antibodies were detected with 1:5000 dilution of murine anti-M13 monoclonal antibody (GE Healthcare, Little Chalfont, UK) and HRP-conjugated goat anti-mouse IgG (Invitrogen, Waltham, Massachusetts, USA).^29^ The substrate solution, TMB (3,3,5,50-tetramethylbenzidine), was added and incubated in the dark for 30 min. The cells were spun down at maximum rate and 100 µL/well of the supernatant was transferred to a new plate which had already been filled with 50 µL of 1 M sulfuric acid per well to stop the color development.^30^ The absorbance was read at 450 nm using a microplate reader (BioTek, Winooski, VT, USA).

### Screening by monoclonal phage ELISA

As a primary screening of the binding specificity, monoclonal phage-scFvs were used for investigation of their binding reactivity to AGS and MKN-45 cells by cell-based ELISA assay. Individual colonies from the third and fourth rounds of panning were randomly picked and transferred to a 96-well cell culture plate containing 100 µL/well of 2xTY-Amp-Glc (2xTY supplemented with 100 µg/mL ampicillin and 1% glucose) and grown overnight by shaking 1000 rpm at 37°C.^27^ The next day, 2 µL of this culture was inoculated to a new plate containing 200 µL/well of 2xTY-Amp-Glc and grown until the OD_600_ reached 0.4.^21^ After infection with KM13 helper phage, the culture medium was altered to 2xTY-Amp-Glc-Kan (supplemented with 100 µg/mL ampicillin, 0.1% glucose, and 50 µg/mL kanamycin) and incubated shaking overnight at 30°C. The scFv-displaying phages rescued into the supernatants of the bacterial cultures were subsequently utilized for ELISA as described above.

### Plasmid extraction, polymerase chain reaction (PCR), and analysis of the clone diversity

The positive clones selected by ELISA were picked into LB-Amp-Glc medium and grown for 18-20 h at 37°C. Plasmid extractions were carried out with the QIAprep^®^ Spin Miniprep Kit (QIAGEN, Hilden, Germany) as stated by the provided manual. PCR was performed using pIT2-specific primers, forward LMB3 and reverse pHEN, which are hybridized to sequences flanking the insert. Reactions were carried out in a total volume of 25 µL at the annealing temperature of 55°C. The PCR products were then evaluated using electrophoresis on 1% agarose gel. The plasmids containing scFv genes with confirmed size were subsequently sequenced using primer LMB3 (CAGGAAACAGCTATGAC) and ABI 3730XL DNA sequencer (Macrogen, Seoul, South Korea). The nucleotide sequences were aligned in VBASE2 (http://www.vbase2.org/vbase2.php) and translated with EditSeq sequence analysis software.

### Whole cell ELISA and flow cytometry

Binding reactivity of the monoclonal phage-scFvs was further verified by cell-based ELISA and flow cytometry using AGS, MKN-45, and NIH-3T3 cells. To this end, phage-scFvs of 4 individual clones were produced in large culture volumes of 2xTY-Amp-Glc medium and purified by PEG (polyethylene glycol) precipitation. For flow cytometry analysis, the cells and the phage-scFvs were blocked in FACS buffer (PBS solution containing 3% BSA and 0.03% NaN_3_) and aliquots containing 5x10^5^ cells were incubated with 100 µL of phage particles (5×10^11^ CFU) for 1 h on ice. The membrane-bound phage particles were subsequently detected by incubation with anti-M13 monoclonal antibody and FITC-conjugated rat anti-mouse IgG (BioLegend, San Diego, California, USA). All incubations were performed at 4°C followed by two washes with PBS containing 1% BSA and 0.03% NaN_3_. After the final wash and resuspension in 500 µL PBS, the samples were investigated through Becton Dickinson FACSCalibur flow cytometer (BD Biosciences, Franklin Lakes, NJ, USA) while dead cells were excluded by adding 5 µL of propidium iodide.

### Statistical analysis

Data were represented as mean ± SD (standard deviation) of duplicate or triplicate experiment for each assay.

## Results and Discussion

### Cell-based selection of the library to enrich AGS-binding phage-scFvs

**Figure 1:**
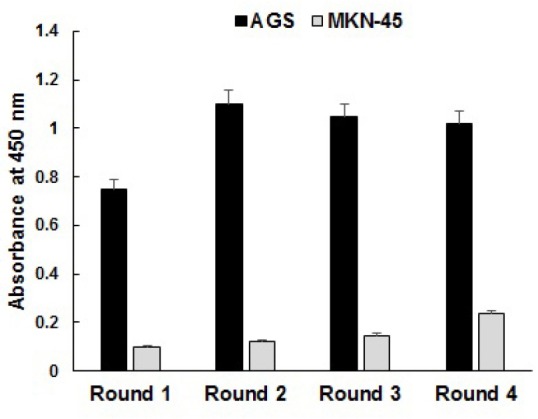
ELISA analysis of the library panned against live AGS and MKN-45cells in suspension. Phage pools obtained after each round of selection were incubated with AGS (positive target cell) and MKN-45 (negative control cell) cells. The values were calculated as absorbance at 450 nm (A450) subtracted from background absorbance at 630 nm. Assays were done in triplicate.

In an attempt to select specific and potentially therapeutic phage antibodies, we employed a subtractive WCP method for isolation of human anti-AGS phage-scFvs from a semisynthetic library, Tomlinson I. The selection procedure involved depletion of the library using NIH-3T3 and MKN-45 cells followed by selection on AGS cells in order to increase the likelihood of isolating phage antibodies that show specificity for the differentiated tumor cells. Four successive rounds of selection and amplification were successfully proceeded to enrich AGS-specific binders. The progressive enrichment of specific binders was monitored by phage titer measurement (as summarized in Table 1) and polyclonal phage ELISA. The reduction of phage bound fraction and enrichment factor in round 3 was probably for decreasing the number of AGS cells to 5×10^6^ in rounds 3 and 4 along with increasing the number of washes to 10 times, both of which were performed to obtain high-affinity clones. Further increase of the bound fraction and enrichment factor in round 4 indicates the amplification of specific binders. Based on the polyclonal phage ELISA results (Figure 1), strong binding reactivity to AGS cells showed enrichment of specific binders. AGS-binding phage-scFvs started to enrich from round 1 toward round 4 upon generating strong positive signals over negative control signals. MKN-45 binders were a minor population within the pool as indicated by lower overall binding signals. Despite altering the number of AGS cells and washes after the second round, the ELISA signals produced by specific binders were roughly constant across the rounds 3 and 4. The ELISA results were not in consistence with the results of phage titration presented in Table 1. This phenomenon could be explained by the fact that the calculation of enrichment factor is based on the count of infective phage particles whereas ELISA signals are mainly produced by binding reactivity of functional phage-displayed scFvs that represent only a minor population of infective phage particles (approximately 1%).

**Table 1:** Results of library selection on whole cells in suspension

**Selection round**	**No. of AGS cells**	**Phage input** **(CFUs)** ^a^	**Phage output (CFUs)** ^b^	**Bound fraction (%recovery)** ^c^	**Enrichmentfactor** ^d^	**Positive rate (%)** ^e^
1	10^7^	10^12^	0.475×10^6^	0.475×10^-4^	-	ND^*^
2	10^7^	0.49×10^12^	1.13×10^9^	0.2306	4854	ND
3	5×10^6^	0.585×10^12^	0.13×10^9^	0.0222	467	5/96
4	5×10^6^	0.917×10^12^	1.05×10^9^	0.1145	2410	19/96

The library was depleted via incubation with NIH-3T3 and MKN-45 cells and subsequently selected on AGS cells. Bound phages were eluted with trypsin and titered in *E. coli* TG1 cells for CFU (colony forming unit) determinations. Phage titer measurements were done in duplicates. The round 1 amplified phage was used as the phage input for round 2 of panning, and so forth. ^a^Total number of CFUs of phage used for each selection round. ^b^Total number of CFUs of phage eluted after each selection round. ^c^(Phage output / Phage input) × 100. ^d^ Fold increase in % phage recovery, compared to the first round of selection. ^e^Number of individual AGS-binding phage-scFv clones per total number of clones screened as determined by monoclonal phage ELISA. ^*^Not determined.

### Screening of individual AGS-specific phage-scFvs

**Figure 2:**
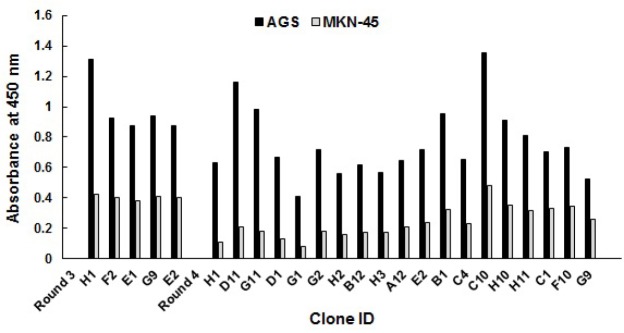
ELISA screening of individual phage-scFv clones. Reactivity of 192 individual phage clones selected from the third and fourth round of panning was tested using live AGS and MKN-45 cells in suspension. Clones were scored positive if ELISA values for the positive cells were at least 2-folds over the negative ones. The values were calculated as absorbance at 450 nm (A450) subtracted from background absorbance at 630 nm.

Screening of 192 individual phage clones from rounds 3 and 4 was performed by whole cell ELISA, as shown in Figure 2. Specific binders were identified by checking their binding reactivity on cells in suspension where the ELISA values for AGS cells were at least 2-folds over those of MKN-45 cells. A total of 24 clones (12%) were considered as positive hints with 5 and 19 clones from rounds 3 and 4, respectively. The highest binding reactivity to AGS cells was found in clones H1, D11, G11, and D1 of round 4.

### PCR analysis and DNA sequencing of the selected clones

The presence of full-length scFv inserts (935 base pairs) in the selected positive clones was demonstrated by PCR analysis. The sequence analysis of the ELISA-positive clones (24 clones) by multiple alignments and CDR (complementarity-determining regions) comparison in VBASE2 (Table 2) indicated 14 unique phage-scFv clones with the highest frequency (12.5%) for clone H1 of round 4. The deduced amino acid sequences confirmed correct in-frame antibody structures except for two clones, C1 of round 4 and H1 of round 3, which represented stop codon mutations in the framework region 1. Moreover, comparison of the amino acid sequences revealed that the majority of clones possessed the highest diversity in CDR3 region of VH and VL and the lowest diversity in VL-CDR2 region.

**Table 2:** Sequence diversity of heavy and light chains for ELISA-positive clones

**Selected clones**	**V**_H_ **chain**			**V**_L_ **chain**			**No. (%)**
	**CDR1 (27-34)**	**CDR2 (56-63)**	**CDR3 (105-113)**	**CDR1 (27-32)**	**CDR2 (56-58)**	**CDR3(105-113)**	
Round 3	H1	GFTFSSYA	ISAYGYNT	AKNYGSFDY	QSISSY	SAS	QQDSSGPAT	-
	F2	GFTFSSYA	IANNGNST	AKSAAGFDY	QSISSY	DAS	QQAYDYPDT	8.3
	E1	GFTFSSYA	ITSTGGGT	AKTYSGFDY	QSISSY	SAS	QQSYDYPDT	8.3
Round 4	H1	GFTFSSYA	IYNDGNYT	AKDSGAFDY	QSISSY	YAS	QQYADNPTT	12.5
	D11	GFAFSSYA	ISASGTDT	AKGASAFDY	QSISSY	AAS	QQDGSDPNT	-
	G11	GFTFSSYA	ITASGNYT	AKNSNSFDY	QSISSY	AAS	QQNYDSPST	-
	D1	GFTFSSYA	ISSSGTST	AKTGSAFDY	QSISSY	NAS	QQAYDAPDT	-
	G1	GFTFSSYA	ISASGTGT	AKTASAFDY	QSISSY	GAS	QQSYDAPET	-
	H2	GFTFSSYA	IYASGAGT	AKNYSTFDY	QSISSY	SAS	QQASSNPTT	12.5
	B12	GFTFSSYA	ITTGGSGT	AKTDSSFDY	QSISSY	NAS	QQAYDYPDT	20.8
	B1	GFTFSSYA	IYASGAGT	AKTDSAFDY	QSISSY	AAS	QQSSDAPDT	-
	C10	GFTFSSYA	IASSGYAT	AKTNAAFDY	QSISSY	NAS	QQAYDYPDT	-
	H10	GFTFSSYA	IGNYGDDT	AKDSGSFDY	QSISSY	NAS	QQSAYTPDT	-
	C1	GFTFSSYA	ITAGGGST	AKSTASFDY	QSISSY	NAS	QQAYSSPDT	-

Multiple alignment of DNA sequences was carried out using VBASE2 and the results are illustrated as CDR regions comparison. Deduced amino acid sequences of CDR regions relating to the selected phage-scFvs are depicted in single-letter amino acid code. Numbering and assignment of CDR regions were according to IMGT.

### Specificity characterization of anti-AGS phage-scFvs

**Figure 3:**
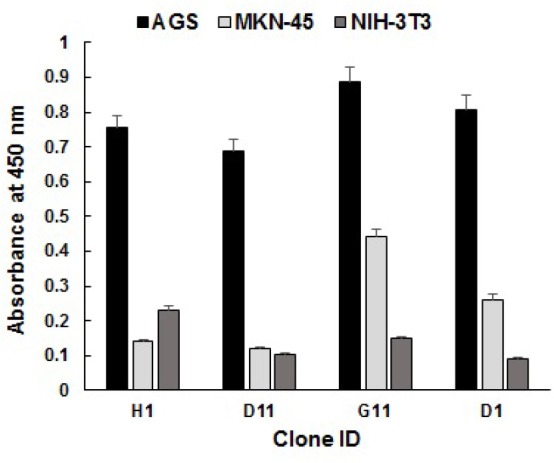
Specificity analysis of PEG-purified monoclonal phage-scFvs using ELISA. Four clones with strong positive signals from the fourth round of panning were produced in large culture volumes and purified by PEGylation. The reactivity of the phage-scFvs was investigated using AGS cells, as positive control, and MKN-45 and NIH 3T3 cells, as negative controls. The values were calculated as absorbance at 450 nm (A450) subtracted from background absorbance at 630 nm. The results are representative of two independent experiments.

**Figure 4:**
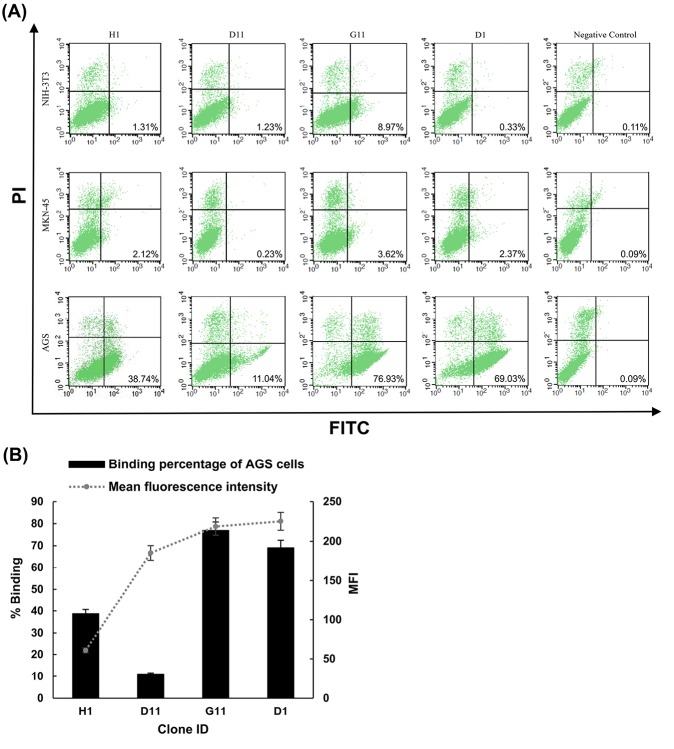
Flow cytometric analysis of phage-scFv binding reactivity. PEG-purified monoclonal phage-scFvs were used to stain AGS (as positive target), and MKN-45 and NIH-3T3 cells (as negative cells). The unselected library I was included as negative control and the experiments were repeated twice. (A) Dead cell and debris were excluded from acquisition and data analysis was performed by gating the cell populations at forward/side scatter dot plot as well as gating the PI-negative cells which are representative of live cell population. Approximately 104 live cells were read out and dot plot analyses were executed by quadrant mark based on the isotype control of each cell line (cells not incubated with phage-scFvs). The lower-right quadrant indicates the percentage of cells that were stained by phage-scFvs (as depicted by clone ID above each plot). (B) Flow cytometry results were also quantified for AGS-positive phage-scFvs in terms of total binding percentage and mean fluorescence intensity (MFI). The results are representative of two independent experiments.

For further verification of the reactivity pattern, four phage-scFv clones from the round 4 of panning with the strong binding signal in the screening were PEG-precipitated and subjected to test against AGS, MKN-45, and NIH-3T3 cells by cell-based ELISA and flow cytometry. As illustrated in Figure 3, the phage-scFv clones bound specifically and significantly to AGS cells and did not show any significant reactivity with MKN-45 and NIH-3T3 cells. To further interrogate the binding specificity, the selected clones were used to stain the live intact cells in flow cytometry. Similarly, all phage-scFv clones showed significant binding reactivity to AGS cells and low overall background staining of MKN-45 and NIH-3T3 cells (Figure 4A). As shown in Figure 4B, the selected clones produced different fluorescent staining patterns of AGS cells in terms of total binding percentage and mean fluorescent intensity (MFI). The highest total percentage and MFI values belonged to clones G11 (76.93% and 218.59) and D1 (69.03% and 225.21) while the lowest values were produced by clones H1 (38.74% and 60.76) and D11 (11.04% and 185.28). The unselected library was used as a negative control and no fluorescence staining of the cells was observed. The flow cytometry results also revealed that the phage-scFv clones probably recognized distinct molecular targets on AGS cells. For example, clones G11 and D1 are possibly specific to an antigen which is overexpressed by most of AGS cells within the population, whereas clone D11 probably recognizes a cell surface protein overexpressed by a minor population. Phage clone H1 probably recognizes a membrane protein with the lowest expression on AGS cells. We speculate that the isolated phage-scFvs have potential implementation for comparative surface proteomic analysis of differentiated (AGS) and poorly differentiated (MKN-45) gastric adenocarcinoma cell lines that can lead to recognition of cell surface proteins mediating proliferation and differentiation of the tumor cells.^3^ One of the limitations of the subtractive WCP method employed here is probably the unavailability of further gastric adenocarcinoma cell lines and normal gastric tissue, both of which could be utilized either for additional specificity analysis or depletion of the library.

The application of phage antibody libraries and subtractive WCP strategy is a simple and fast way to isolate immunoreagents directed against cellular membrane biomarkers. So far, several studies have employed phage libraries and WCP approach for target identification. For instance, Sharma et al^31^ successfully employed a direct WCP method to screen Tomlinson library against cell surface epitopes expressed on ovarian cancer cells. Proper discriminative reactivity of the selected scFvs was exhibited for the target cells, normal ovarian cell line, and a panel of tumors. In another attempt, a novel platform for tumor antigen discovery was developed based on a subtractive WCP scheme through screening a single domain antibody library against lung adenocarcinoma cells and resulted in identifying the cognate antigens.^32^ Therefore, such antibody fragments can be utilized as valuable probing molecules for mapping the variations in gene expression between two populations of cells such as differentiated and undifferentiated cells or transformed and non-transformed cells.

Decoding the human proteome has become an essential step in discovering novel biomarkers which play a central role in diseased conditions.^33^ The exploration of disease-related biomarkers previously depended on the data obtained from transcriptome, but levels of mRNA do not always correlate with the respective cell surface protein. Therefore, current technologies for human proteome profiling are mainly based upon the application of mass spectrometry,^34,35^ affinity proteomics platforms,^36^ or a combination of both.^37^ Affinity-based approaches rely upon the utilization of various types of affinity molecules including conventional monoclonal antibodies (mAbs), recombinant antibody fragments, as well as non-antibody reagents (e.g., aptamers and affibodies) that function as protein-specific probes to discern and quantify molecular targets in their natural milieu. Surface epitope profiling based on mAbs is capable of recognizing post-translational or any other molecular modifications of proteins which are overexpressed in malignancies. PDT has emerged as a significant tool for manufacturing research, diagnostic, and therapeutic antibodies with desired characteristics. PDT with an advantage of compatibility to automation provides a proficient choice for a proteome-wide mAb project. One of the most significant requirements of these library-selected mAbs is the ability to distinguish between the proteome of different cells and tissues, i.e., healthy and malignant cells. In this respect, using whole cells as a complex source of target antigen (known as WCP) is a preferred alternative for selection of functional antibodies recognizing molecular targets with higher specificity because whole intact cells maintain the native conformational structure of membrane proteins as close as to those in the *in vivo* state. By arraying mAbs through WCP and parallel screening on diverse cells or tissues, the isolation of antibodies that recognize differently expressed proteins will be achievable. Then, the selected antibodies that bind to cell surface proteins found in one tissue but not the other could be used to characterize the respective differently expressed proteins which probably play a critical role in pathophysiological processes such as proliferation, differentiation, and migration of cells.

## Conclusion

Generation of binders capable of differentiating between malignant and healthy cells is essential for identification and validation of gene products that may serve as novel molecular targets. Procedures that allow for targeting membrane proteins in their native conformation may give rise to the identification of molecules involved in the development of tumor tissues. In the present study, PDT was applied to identify phage-scFvs, which could discriminate AGS cells from MKN-45 and normal NIH-3T3 cells. We employed a subtractive WCP method for isolating human anti-AGS phage antibodies from a semisynthetic library, Tomlinson I. The selection procedure involved depletion of the library with NIH-3T3 and MKN-45 cells followed by selection on AGS cells in order to increase the likelihood of isolating antibodies that show specificity for the differentiated tumor cells. In the future study, we intend to identify the cognate antigens of the scFvs through immunoprecipitation of AGS cell proteome and interrogate the function of the scFvs in targeted delivery of bioactive moieties such as drugs, toxins, cytokines, and radionuclides to cancer sites.

## Ethical Issues

This study was approved by the Ethics Committee at Tabriz University of Medical Sciences. Ethical code: 5/D/1039964.

## Conflict of Interest

The authors declare no conflicts of interest.

## Acknowledgments

The authors appreciate the technical support provided by colleagues at Research Center for Pharmaceutical Nanotechnology at Tabriz University of Medical Sciences. The study was funded by the postgraduate grant from Research Center for Pharmaceutical Nanotechnology (Grant no: 95011). This work was done as an MSc thesis written by Sepideh Nikfarjam at Tabriz University of Medical Sciences.
